# Influence of *HRH2* promoter polymorphism on aberrant DNA methylation of *DAPK* and *CDH1* in the gastric epithelium

**DOI:** 10.1186/1471-230X-13-1

**Published:** 2013-01-02

**Authors:** Tomoe Nomura, Tomomitsu Tahara, Hisakazu Shiroeda, Takahiro Minato, Yasuhiro Matsue, Ranji Hayashi, Kazuhiro Matsunaga, Toshimi Otsuka, Masakatsu Nakamura, Nobuyuki Toshikuni, Tomoyuki Shibata, Tomiyasu Arisawa

**Affiliations:** 1 Department of Gastroenterology, Kanazawa Medical University, 1-1, Daigaku, Uchinada-machi, Ishikawa, 920-0293, Japan; 2 Department of Gatroenterology, Fujita Health University, School of Medicine, 1-98, Dengakugakubo, Kutsukake-cho, Toyoake, 470-1192, Japan

**Keywords:** *HRH2*, Genetic polymorphism, Aberrant DNA methylation

## Abstract

**Background:**

Aberrant methylation patterns in CpG island are known to be influential in gene silencing. Histamine plays important physiological roles in the upper gastrointestinal tract and acts via the H2 receptor. We report an investigation into the effect of *HRH2* promoter polymorphism (rs2607474 G > A) on the methylation of *DAPK* and *CDH1*.

**Methods:**

Non cancerous gastric mucosa samples were obtained from 115 subjects with gastric cancer (GC) and 412 non-cancer subjects (non-GC). Methylation status of genes was determined by MSP. The genotyping of rs2607474 was performed by PCR-SSCP.

**Results:**

Methylation of *DAPK* and *CDH1* was observed in 296 and 246 subjects, respectively. The frequency of *CDH1* methylation in the subjects with GC was significantly lower in cancer lesion than in non cancerous mucosa, whereas that of *DAPK* methylation was not different. The allelic distribution of rs2607474 was 401GG, 119GA and 7AA. The GG homozygote was associated with a significantly increased risk for methylation of both *DAPK* and *CDH1* (p < 0.0001 and p = 0.0009, respectively). In the non-GC subjects or more than 60 years of age, GG homozygote was more closely associated with both *DAPK* and *CDH1* methylation. However, this genotype did not show an increased risk for the development of methylation of both genes in patients with GC. In *H. pylori* negative subjects, GG homozygote showed an increased risk for the methylation of both *DAPK* and *CDH1* (p = 0.0074 and p = 0.0016, respectively), whereas this genotype was associated with an increased risk for the development of *DAPK* methylation in *H. pylori* positive subjects (p = 0.0018). In addition, in subjects older than 60 years of age, atrophy and metaplasia scores were significantly higher in the GG homozygote (p = 0.011 and p = 0.039, respectively) and a significant correlation was observed between age and atrophy or metaplasia.

**Conclusions:**

Our results suggest that rs2607474 GG homozygote confers a significantly increased risk for age- and inflammation-related *DAPK* and *CDH1* methylation.

## Background

Gastric cancer is one of the most common cancers worldwide and is associated with a high mortality rate [1,2]. Several cancers, including gastric tumors, show methylation of multiple genes including E-cadherin (*CDH1*)*,* death-associated protein kinase (*DAPK*) and cyclin-dependent kinase inhibitor 2A (*CDKN2A*) [3,4]. Methylation of promoter CpG islands leads to DNA structural changes and, consequentially, gene inactivation [5]. Many studies have identified this silencing by DNA methylation as a mechanism responsible for tumor suppressor inactivation. However, some genes are also methylated in non-neoplastic tissues due to ageing [6,7], and it has also been suggested that gene methylation occurs during chronic inflammation in various tissues [8-10]. In gastric mucosa, it has been postulated that methylation of CpG island is induced by *Helicobacter pylori* (*H. pylori*) infection in non-cancerous mucosa [11,12] and considered as the precancerous conditions in gastric carcinogenesis [13]. Among several genes, *DAPK*, *CDH1*and *CDKN2A* are known to be frequently methylated in non-neoplastic gastric mucosa and this methylation is linked to age, *H. pylori* infection, histological degree of gastritis, and gastric cancer [11,13,14].

However, histamine, one of the active amines released in response to a variety of physiological stimuli, is widely distributed in the gastrointestinal tract including the stomach and is involved in the pathogenesis of gastro-duodenal ulceration and gastric inflammation [15]. In the stomach, H2 receptors, although widely distributed in body tissues, seem to have a central role in the regulation of acid secretion, as confirmed by the widespread use of H2 receptor blockers in the therapy of acid-related disorders [16,17]. Histamine plays an important role in gastric inflammation acting via the H2 receptor, although *H. pylori* infection is one of the major contributing factors to the development of gastro-duodenal inflammation [18]. Recently, the association between genetic polymorphisms of histamine receptor genes and susceptibility to schizophrenia, and its clinical response to clozapine treatment has been studied [19]. This investigation of the polymorphisms of *HRH2*, coding for the H2 receptor, reveals association between the genotype at the *HRH2* -1018 G/A locus (rs2607474) and clinical response to clozapine treatment. In addition, this report has shown that rs2607474 is located in an enhancer element of the *HRH2* gene promoter [19,20] and it is thus likely that this variant induces changes in the expression of receptors. According to HapMap-JPT, there is only one linkage disequilibrium (LD) block, composed of rs686874, rs2067474, rs678591, rs645574, rs2890892 and rs11954815, in HRH2. All the other SNPs are minor polymorphisms. Therefore, we speculated that this LD block influence the expression and/or function of histamine H2 receptor and selected rs2607474 as a Tag SNP. There has not yet been any report on the rs2607474 affect on the development and progression of gastrointestinal disorders. We hypothesized that the rs2607474 may influence the development of aberrant DNA methylation patterns in gastric mucosa.

In the present study, we investigated the relationship between *HRH2* promoter polymorphism (rs2607474) and DNA methylation of *DAPK* and *CDH1* in non-cancerous gastric epithelium. The difference of DNA methylation status among gastric cancer patients and non-cancerous subjects was also investigated.

## Methods

### Tissue samples, DNA extraction, and helicobacter pylori infection status

Our studied population comprised 527 subjects (412 without malignant tumors and 115 with gastric cancer) recruited from the Endoscopy Center of Fujita Health University Hospital or Kanazawa Medical University Hospital. Patients with systemic severe diseases were excluded from this study. Those with active gastric or duodenal ulcers were also excluded. All subjects underwent upper endoscopy with biopsy from non-cancerous mucosa. In 115 subjects with gastric cancer, biopsy specimen was also obtained from cancer lesion. Parts of each specimen were immediately frozen and stored at −80°C, while some of the other part was fixed in 10% buffered formalin and embedded in paraffin. Later, Genomic DNA was extracted directly from frozen specimens using standard phenol/chloroform method after proteinase K digestion. In 296 of 412 subjects without gastric cancer, the severity of chronic gastritis was classified according to the updated Sydney system [21] by a pathologist who had no access to any clinical information. *H. pylori* infection status was assessed by serology, histology, or urea breath test. Subjects were diagnosed as infected when at least one of the diagnostic tests was positive.

The Ethics Committees of the Fujita Health University and Kanazawa Medical University approved the protocol, and prior, written informed consent was obtained from all participating subjects.

### Bisulfite modification and methylation-specific PCR (MSP)

To examine DNA methylation, genomic DNA was treated with sodium bisulfite using BislFast DNA Modification Kit for Methylated DNA Detection (TOYOBO, Co., Ltd., Osaka, Japan). MSP for *DAPK* and *CDH1* were carried out using the methods reported by Katzenellenbogen et al. [22] and Herman et al. [23], respectively.

In brief, MSP reactions were carried out with the following primer pairs using EX Taq HS (Takara Bio, Inc., Shiga, Japan).

Primer pairs:

DAPK: methylated forward; 5^′^- ggatagtcggatcgagttaacgtc-3^′^

reverse; 5^′^- ccctcccaaacgccga-3^′^,

DAPK: unmethylated forward; 5^′^- ggaggatagttggattgagttaatgtt-3^′^,

reverse; 5^′^- caaatccctcccaaacaccaa-3^′^,

CDH1: methylated forward; 5^′^- ttaggttagagggttatcgcgt-3^′^,

reverse; 5^′^- taactaaaaattcacctaccgac-3^′^,

CDH1: unmethylated forward; 5^′^- taattttaggttagagggttattgt-3^′^,

reverse; 5^′^- cacaaccaatcaacaacaca-3^′^,

Annealing temperature and times were determined using DNA from peripheral blood of a young individual without *H. pylori* infection as a negative control and this DNA methylated with SssI methylase (NEW ENGLAND BioLabs Inc., Beverly, MA) as a positive control. The MSP was carried out in a volume of 20μL containing 0.1 μg of bisulfite-modificated DNA. The DNA was denatured at 95°C for 5 minutes, followed by 35 cycles at 95°C for 30 seconds, 64 or 65°C (for *CDH1* or *DAPK*, respectively) for 1 minutes, and 72°C for 1 minute with a final extension at 72°C for 5 minutes. The bands of MSP were detected by electrophoresis in 3.0% agarose gels stained with ethdium bromide. Hypermethylation was defined as the presence of a positive methylation band showing signals approximately equal to or greater than that of the size marker (10 ng/μL: 100 bp DNA Ladder, Takara Bio, Inc., Shiga, Japan) regardless of the presence of unmethylated bands [4].

### Genotyping of HRH2 polymorphism

The rs2607474 was genotyped by PCR-SSCP as reported previously [24,25]. To detect the genotype, using the primer pair (forward: 5^′^-acctgacccttttctgaaaaagtttgtc-3^′^, and reverse: 5^′^-ctactcctctgaagtgctgagaaccat-3^′^), PCR was carried out in a volume of 20 μL containing 0.1 μg of genomic DNA. The DNA was denatured at 95°C for 3 minutes, followed by 35 cycles at 96°C for 15 seconds, 60°C for 30 seconds, and 72°C for 30 seconds, with final extension at 72°C for 5 minutes. Thereafter, 2 μL of PCR product was denatured with 10 μL of formamide (Sigma-Aldrich Co., St. Louis, USA) at 95°C for 5 minutes. SSCP was carried out at 6°C using a GenePhor DNA separation system with GeneGel Excel 12.5/24 (GE Healthcare, USA), after which the denatured single strand DNA bands were detected using a DNA Silver Staining Kit (GE Healthcare). We confirmed that single strand DNAs were clearly separated by this condition [25]. SSCP results were confirmed using positive DNA fragments prepared by nested-PCR as reported previously [24,26].

### Statistical analysis

Age and updated Sydney system scores were expressed as mean ± SD. Mean ages between two groups were compared with Student’s *t* test. The ratio of gender, *H. pylori* infection and DNA methylation were compared using Fisher’s exact test. The allele counts were also compared between the methylated and the unmethylated groups by Fisher’s exact test. Adjusted ORs were calculated with the use of logistical regression analysis after adjustment for age, gender and *H. pylori* infection status. Each updated Sydney system scores among 2 groups was compared by Mann Whitney *U*-test. The correlation between age and each updated Sydney system score was assessed by ANOVA. For all analyses, the level of significance was set at *p* < 0.05.

## Results

### Subjects

A total of 412 non-cancer (non-GC) subjects and 115 gastric cancer (GC) patients participated in this study. These characteristics are summarized in Table 1. The mean age and male/female ratio of the GC group were significantly higher than those in the non-GC group. The *H. pylori* positive ratio of the GC group was also significantly higher than that of the non-GC group. Furthermore, the frequencies of CpG methylation of both *DAPK* and *CDH1* were significantly higher in the GC group than in the non-GC group.

**Table 1:** Characteristics of the subjects and frequencies of gene methylation

	**Overall**	**non-GC**	**GC**	p value*
the number of subjects	527	412	115	
mean age ± SD	61.0 ± 13.7	60.0 ± 13.8	66.2 ± 9.7	0.0019
male : female	319 : 208	235 : 177	84:31	0.0018
*H.pylori positive ratio*	338/527	249/412	89/115	0.00091
DAPK methylated ratio	296/527	201/412	95/115	<0.0001
CDH1 methylated ratio	246/527	150/412	96/115	<0.0001

*: non-GC group vs. GC group.

### Frequencies of rs2607474 genotypes among DAPK methylated and unmethylated groups

The distribution of this genotype in all 527 subjects was 401GG, 119GA and 7AA and was in Hardy-Weinberg equilibrium (p = 0.70). The mean age, *H. pylori* positive ratio and GC/non-GC ratio were significantly higher in the *DAPK* methylated than in the unmethylated groups, whereas the male/female ratio among the two groups was not significantly different (Table 2). The minor allele frequency of rs2607474 in the methylated group was significantly lower than that in the unmethylated group (p < 0.0001, setting α = 0.05, 1-βpower = 0.978). In addition, the frequency of the GG homozygote was significantly higher in the methylated group than in the unmethylated group (p < 0.0001).

**Table 2:** The frequencies of genotypes among DAPK methylated and unmethylated groups

	**DAPK unmethylated**	**DAPK methylated**	p value*
the number of subjects	231	296	
mean age ± SD	59.6 ± 13.4	62.1 ± 13.8	0.035
male : female	137 : 94	182 : 114	NS
*H. pylori positivity*	122/231	216/296	<0.0001
non-GC : GC	211 : 20	201 : 95	<0.0001
HRH2 rs2607474 genotype			
GG	155	246	<0.0001#
GA	72	47	
AA	4	3	
A allele frequency	17.3%	9.0%	<0.0001

*: unmethylated vs. methylated, #: frequency of GG homozygote.

NS: not significant.

### Frequencies of rs2607474 genotypes among CDH1 methylated and unmethylated groups

The *H. pylori* positive ratio and GC/non-GC ratio were significantly higher in the *CDH1* methylated group than in the unmethylated group, whereas mean age and male/female ratio among the two groups were not significantly different (Table 3). The minor allele frequency of rs2607474 in the methylated group was significantly lower than that in the unmethylated group (p = 0.0004, setting α = 0.05, 1-βpower = 0.946) and the frequency of the GG homozygote was significantly higher in the methylated group than in the unmethylated group (p = 0.013).

**Table 3:** The frequencies of genotypes among CDH1 methylated and unmethylated groups

	**CDH1 unmethylated**	**CDH1 methylated**	p value*
the number of subjects	281	246	
mean age ± SD	60.6 ± 14.0	61.4 ± 13.3	NS
male : female	165 : 116	154 : 92	NS
*H. pylori positivity*	152/281	186/246	<0.0001
non-GC : GC	262 : 19	150 : 96	<0.0001
HRH2 rs2607474 genotype			
G/G	197	204	0.013#
G/A	78	41	
A/A	6	1	
A allele frequency	16.0%	8.7%	0.0004

*: unmethylated vs. methylated, #: frequency of GG homozygote.

NS: not significant.

### Risk associated with rs2607474 GG homozygote for DAPK and CDH1 methylation

Overall, rs2607474 GG homozygote conferred a highly significant increased risk for the development of *DAPK* methylation (OR, 2.43; 95%CI, 1.60-3.69; p < 0.0001; Table 4). In the non-GC subjects, the GG homozygote also showed a significantly increased risk for the development of *DAPK* methylation (OR, 2.61; 95%CI, 1.62-4.20; p < 0.0001). However, it displayed neither a significant risk in non cancerous mucosa nor in cancer lesion in subjects with GC (Table 4). The frequency of *DAPK* methylation was not significant different between non cancerous mucosa and cancer lesion in subjects with GC (95/115 vs. 104/115, p = 0.12).

**Table 4:** Risk of −1018 GG homozygote for the development of DAPK methylation

**Overall (527)**	**GG**	**GA**	**AA**	**GG vs A carrier; OR (95%C.I.)**	**p value**
unmethylated (231)	155	72	4	reference	-
methylated (296)	246	47	3	2.43 (1.60-3.69)	<0.0001
non-GC (412)					
unmethylated (211)	138	69	4	reference	-
methylated (201)	167	31	3	2.61 (1.62-4.20)	<0.0001
GC (115)					
(non cancerous mucosa)					
unmethylated (20)	17	3	0	reference	-
methylated (95)	79	16	0	0.825 (0.379-3.73)	0.78
(cancer lesion)					
unmethylated (11)	10	1	0	reference	-
methylated (104)	86	18	0	0.598 (0.069-5.19)	0.64

by logistic regression analysis after adjustment for age, gender and H. pylori infection status.

(): the number of subjects.

On the other hand, rs2607474 GG homozygote was associated with a significantly increased risk for the development of *CDH1*, as well as *DAPK*, methylation (OR, 2.07; 95%CI, 1.35-3.17; p = 0.0009; Table 5). In the non-GC subjects, the GG homozygote was also associated with a significantly increased risk for the development of *CDH1* methylation (OR, 2.25; 95%CI, 1.35-3.75; p = 0.0019). However, it exhibited neither a significant risk in non cancerous mucosa nor in cancer lesion in subjects with GC (Table 5). The frequency of *CDH1* methylation in the subjects with GC was significantly lower in cancer lesion than in non cancerous mucosa (78/115 vs. 96/115, p = 0.009).

**Table 5:** Risk of −1018 GG homozygote for the development of CDH1 methylation

**Overall (527)**	**GG**	**GA**	**AA**	**GG vs A carrier; OR (95%C.I.)**	**p value**
unmethylated (281)	197	78	6	reference	-
methylated (246)	204	41	1	2.07 (1.35-3.17)	0.0009
non-GC (412)					
unmethylated (262)	180	76	6	reference	-
methylated (150)	125	24	1	2.25 (1.35-3.75)	0.0019
GC (115)					
(non cancerous mucosa)					
unmethylated (19)	17	2	0	reference	-
methylated (96)	79	17	0	0.579 (0.120-2.80)	0.50
(cancer lesion)					
unmethylated (37)	30	7	0	reference	-
methylated (78)	66	12	0	1.50 (0.507-4.42)	0.47

by logistic regression analysis after adjustment for age, gender and H. pylori infection status.

(): the number of subject.

### Risk associated with rs2607474 GG homozygotes for the development of DNA methylation in subjects above and below 60 years of age

In subjects younger than 60 years of age, rs2607474 GG homozygote conferred a slightly, but significantly, increased risk for *DAPK* methylation (OR, 2.12; 95%CI, 1.13-3.98; p = 0.020, Table 6), whereas not for *CDH1* methylation. However, the GG homozygote showed a significantly increased risk for the development of both *DAPK* and *CDH1* methylation in subjects older than 60 years (OR, 2.72; 95%CI, 1.55-4.80; p = 0.0005 and OR, 2.81; 95%CI, 1.53-5.17; p = 0.0009, respectively, Table 6).

**Table 6:** Risk of rs2697474 GG homozygote in younger or older subjects

**DAPK methylation**					
= < 60 (233)	GG	GA	AA	GG vs GA + AA; OR (95%CI)	p value
unmethylated (115)	80	32	3	reference	-
methylated (118)	96	21	1	2.12 (1.13-3.98)	0.020
60 < (294)					
unmethylated (116)	75	40	1	reference	-
methylated (178)	150	26	2	2.72 (1.55-4.80)	0.0005
CDH1 methylation					
= < 60 (233)	GG	GA	AA	GG vs A carrier; OR (95%C.I.)	p value
unmethylated (121)	88	29	4	reference	-
methylated (112)	88	24	0	1.55 (0.827-2.91)	0.17
60 < (294)					
unmethylated (160)	109	49	2	reference	-
methylated (134)	116	17	1	2.81 (1.53-5.17)	0.0009

by logistic regression analysis after adjustment for age, gender and H. pylori infection status.

(): the number of subjects.

### Risk associated with rs2607474 GG homozygotes for the development of DNA methylation in H. pylori negative or positive subjects

In *H. pylori* negative subjects, rs2607474 GG genotype was associated with an increased risk for the methylation of both *DAPK* and *CDH1* (OR, 2.70; 95%CI, 1.31-5.57; p = 0.0074, and OR, 4.43; 95%CI, 1.76-11.1; p = 0.0016, respectively, Table 7). However, the GG homozygote displayed an increased risk for the development of *DAPK* methylation in *H. pylori* positive subjects (OR, 2.29; 95%CI, 1.36-3.86; p = 0.0018), but not for *CDH1* methylation.

**Table 7:** Risk of rs2607474 GG homozygote in H. pylori positive or negative subjects

**DAPK methylation**					
*H. pylori negative (189)*	GG	GA	AA	GG vs GA + AA; OR (95%CI)	p value
unmethylated (109)	73	35	1	reference	-
methylated (80)	67	12	1	2.70 (1.31-5.57)	0.0074
*H. pylori positive (338)*					
unmethylated (122)	82	37	3	reference	-
methylated (216)	179	35	2	2.29 (1.36-3.86)	0.0018
CDH1 gene methylation					
H. pylori negative (189)	GG	GA	AA	GG vs A carrier; OR (95%C.I.)	p value
unmethylated (129)	86	41	2	reference	-
methylated (60)	54	6	0	4.43 (1.76-11.1)	0.0016
H. pylori positive (338)					
unmethylated (152)	111	37	4	reference	-
methylated (186)	150	35	1	1.50 (0.900-2.51)	0.12

by logistic regression analysis after adjustment for age and gender.

(): the number of subjects.

### Association between Sydney system scores and rs2607474

**Figure 1:**
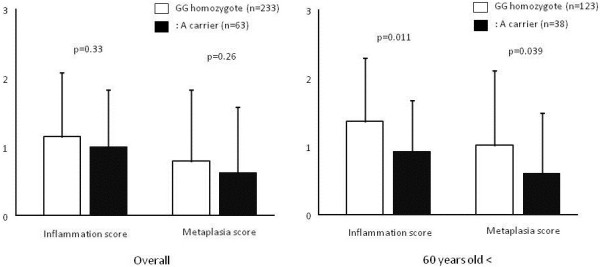
**Comparisons of Sydney system scores among GG homozygote and GA + AA genotype.** Overall, neither atrophy nor metaplasia scores were significantly different among GG homozygote and GA + AA genotype. However, in the subjects older than 60 years, both scores were significantly higher in GG homozygote than in GA + AA genotype.

Overall, there were no significant differences in atrophy or metaplasia scores between the GG homozygote and the GA + AA genotype (Figure 1). However, in subjects older than 60 years of age, both scores were significantly higher in the GG homozygote than in the GA + AA genotype.

**Figure 2:**
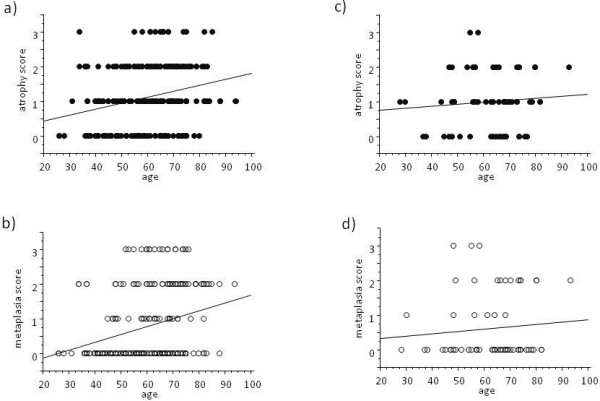
**Association between rs2607474 and gastric mucosal atrophy**. **a:** Correlation between age and atrophy score in GG homozygote Atrophy score was significantly correlated to the age (p = 0.0001 by ANOVA, n = 233). **b:** Correlation between age and metaplasia score in GG homozygote Metaplasia score was significantly correlated to the age (p < 0.0001 by ANOVA). **c:** Correlation between age and atrophy score in GA + AA genotype. There was no significant correlation (p = 0.47 by ANOVA, n = 63). **d:** Correlation between age and metaplasia score in GA + AA genotype. There was no significant correlation (p = 0.48 by ANOVA).

Both atrophy and metaplasia scores were strongly correlated with age in the GG homozygote (both p < 0.0001 by ANOVA, Figure 2a, b), whereas there was no such correlation between age and either score in the GA + AA genotype (p = 0.76 and p = 0.69, respectively, Figure 2c, d).

## Discussion

We have previously reported that CpG island methylation of both *DAPK* and *CDH1*, in non-neoplastic gastric mucosa, corresponded to an increased risk of gastric cancer [4]. In the present study, the frequency of methylation in both genes was significantly higher in the subjects with GC than without GC. However, the frequency of *CDH1* methylation in cancer lesion was significantly lower than that in non cancerous mucosa in the subjects with GC, although the frequency of *DAPK* methylation was not. From these findings, *CDH1* methylation seems to take little part in the development of gastric cancer. In our studied subjects, gene methylation may not reflect the expression of protein or mRNA, although some previous studies demonstrate that these gene methylations contribute to decreasing levels of protein and mRNA [27-30]. Another possibility is that tumor may develop through E-cadherin independent pathways for carcinogenesis. The mechanism of carcinogenesis via gene methylation is still unclear. However, we pay attention to the fact that accumulation of gene methylation actually shows an increased risk of carcinogenesis as many studies have indicated [3,4,6,7,11-14,31,32], even if methylation of *CDH1* may not directly affect the tumor development.

There are few reports that demonstrate an influence of polymorphisms of *HRH2* on the risk for human disorders, but those there are report no association between rs2607474 and neurological or psychological disorders, such as schizophrenia or Parkinson’s disease [19,20,33]. We are unaware of any previous reports on the association between this polymorphism and gastric disorders.In the present study, we investigated the association between rs2607474 and the developments of CpG island methylation of both *DAPK* and *CDH1* in non-cancerous gastric mucosa. Our current study reveals that rs2607474 GG genotype was positively associated with the development of CpG island methylation of both genes. In addition, we also reveal that gastric mucosal atrophy is more severe in comparatively older GG homozygote than GA + AA genotype, and that gastric mucosal atrophy progresses with age only in GG homozygote.

One of the most important factors causing gene methylation in the stomach is *H. pylori* infection [4]. Infection with *H. pylori* first induces chronic superficial gastritis, which can progress to chronic atrophic gastritis, intestinal metaplasia, and dysplasia that can lead gastric carcinoma [34]. The factors promoting *H. pylori*-mediated gastric atrophy are somewhat more controversial. *H. pylori* infection results in an elevation of serum gastrin level, in the early stage of infection, and proceeds to the development of atrophic gastritis. The majority of clinical studies, however, have accepted that proton pump inhibitors (PPIs), which induce achlorhydria and hypergastrinemia, accelerate the onset of atrophic gastritis in *H. pylori* positive patients [35-37]. Therefore, hypergastrinemia seems to promote the gastric mucosal atrophy that is influenced by *H. pylori* infection. Interstingly, long-term treatment of rats and mice with loxtidine, a potent H2 receptor antagonists inducing ECL cell hyperplasia, (as does omeprazole), did not result in loss of parietal cells, but instead appeared to result in increased parietal cells [38,39]. In addition, HDC knockout mice kept on a low-histamine diet showed an expanded parietal cell pool despite exhibiting marked hypergastrinemia [40]. These results suggest that it is the up-regulation of histamine, not achlorhydria nor hypergastrinemia, that contributes to the gradual down-regulation of parietal cell number and gastric atrophy. In our present study, gastric mucosal atrophy progressed with age in rs2607474 GG homozygote cohort, whilst in the GA + AA genotype it did not. In addition, the degree of gastric mucosal atrophy was higher in older subjects who were the GG homozygote than in those that were the GA + AA genotype. In our previous study, a correlation between intestinal metaplasia and gene methylation of various genes has been confirmed [4]. We therefore suggest that gene methylation progresses more rapidly in parallel with gastric mucosal atrophy in the GG homozygote than in the GA + AA genotype. Furthermore, it is possible that the action of histamine is up-regulated in GG homozygote. However, there have been no studies for the effects of rs2607474 on function or expression of H2 receptors and our genetic statistical study did not reveal them. This is one of the limitations in our study. Further study using other methods and other techniques are necessary to evaluate the function of rs2607474.

It has been recently reported that GC patients show higher frequencies of aberrant CpG island methylation in non neoplastic mucosa than non-GC subjects [32]. In our present study, the frequencies of *DAPK* and *CDH1* methylation were also significantly higher in subjects with GC than without GC. On the other hand, rs2607474 GG homozygote was associated with a significantly increased risk for both *DAPK* and *CDH1* methylation in subjects without GC, whereas it wasn’t in subjects with GC. The methylation frequency of *DAPK* and *CDH1* was higher in GC patients than in non-GC cases, and there were few GC patients without methylation of *DAPK* and *CDH1* (20/115 and 19/115, respectively). That is, almost all GC patients have non-cancerous gastric mucosa in which some genes are already methylated. In addition, the cohort composed of only GC patient may have a certain kind of bias. Therefore, the influence of *HRH2* genotype on methylation status of non-cancerous mucosa in patients who have already developed gastric cancer may be indistinct.

A significantly increased risk of the GG homozygote for *DAPK* methylation was seen regardless of the age or *H. pylori* infection status, whilst that for *CDH1* was not seen in the comparatively younger subjects or *H. pylori* positive subjects. Why such a difference was seen is unknown. The mechanisms which ageing or inflammation influences methylation are unclear. We demonstrated in this study that, in the GG homozygote, gastric mucosal atrophy was correlatively progressed with age and was more severe in the comparatively older subjects. As for one possibility, it might be hard for *CDH1* methylation to undertake the influence of ageing or inflammation than *DAPK* methylation.

## Conclusions

3In conclusions, the *HRH2* promoter polymorphism rs2607474 influences the methylation status of *DAPK* and *CDH1*. These findings are observed in non-GC subjects, not in GC patients. The rs2275913 GG homozygote confers a significantly increased risk for age- and inflammation-related gene methylation of both genes.

## Abbreviations

ANOVA: Analysis of variance; *CDH1*: E-cadherin gene; *CDKN2A*: Cyclin-dependent kinase inhibitor 2A gene; CI: Confidence interval; *DAPK*: Death-associated protein kinase gene; GC: Gastric cancer; *H. pylori*: Helicobacter pylori; *HRH2*: Histamine H2 receptor gene; MSP: Methylation-specific PCR; OR: Odds ratio; PG: Pepsinogen; SSCP: Single Strand Conformation Polymorphism; SD: Standard deviation.

## Competing interests

The authors declare that they have no competing interests.

## Authors’ contributions

TA analyzed the data, wrote the paper and was responsible for the conception of the study and designed the study. TT participated in the design of the study. TN, together with HS, TM, YM, RH, KM, TO, MN, NT and TS obtained the samples and the data. All authors approved of the final manuscript prior to submission.

## Pre-publication history

The pre-publication history for this paper can be accessed here:

http://www.biomedcentral.com/1471-230X/13/1/prepub
